# Spin ballet for sweet encounters: saturation-transfer difference NMR and X-ray crystallography complement each other in the elucidation of protein–glycan interactions

**DOI:** 10.1107/S2053230X18006581

**Published:** 2018-07-26

**Authors:** Bärbel S. Blaum, Ursula Neu, Thomas Peters, Thilo Stehle

**Affiliations:** a Interfaculty Institute of Biochemistry, University of Tübingen, 72076 Tübingen, Germany; b Department of Biomolecular Systems, Max Planck Institute of Colloids and Interfaces, 14424 Potsdam, Germany; c Institute of Chemistry and Metabolomics, University of Lübeck, 23562 Lübeck, Germany; d Department of Pediatrics, Vanderbilt University School of Medicine, Nashville, Tennessee, USA

**Keywords:** saturation-transfer difference NMR, STD-NMR, polyomavirus, carbohydrates, lectins, structural biology

## Abstract

Biomolecular NMR spectroscopy has limitations in the determination of protein structures: an inherent size limit and the requirement for expensive and potentially difficult isotope labelling pose considerable hurdles. Therefore, structural analysis of larger proteins is almost exclusively performed by crystallography. However, the diversity of biological NMR applications outperforms that of any other structural biology technique. For the characterization of transient complexes formed by proteins and small ligands, notably oligosaccharides, one NMR technique has recently proven to be particularly powerful: saturation-transfer difference NMR (STD-NMR) spectroscopy. STD-NMR experiments are fast and simple to set up, with no general protein size limit and no requirement for isotope labelling. The method performs best in the moderate-to-low affinity range that is of interest in most of glycobiology. With small amounts of unlabelled protein, STD-NMR experiments can identify hits from mixtures of potential ligands, characterize mutant proteins and pinpoint binding epitopes on the ligand side. STD-NMR can thus be employed to complement and improve protein–ligand complex models obtained by other structural biology techniques or by purely computational means. With a set of protein–glycan interactions from our own work, this review provides an introduction to the technique for structural biologists. It exemplifies how crystallography and STD-NMR can be combined to elucidate protein–glycan (and other protein–ligand) interactions in atomic detail, and how the technique can extend structural biology from simplified systems amenable to crystallization to more complex biological entities such as membranes, live viruses or entire cells.

## Abstract 2

Proton-based saturation-transfer difference NMR (STD-NMR) is a technique that has proven to be particularly useful in the elucidation of protein–glycan interactions. This article explains the method for non-NMR spectroscopists and uses a number of crystal structures of viral capsid proteins in complex with respective glycan receptors to compare findings from crystallography and STD-NMR side by side, highlighting the complementary nature of the approaches.

## 1. Introduction   

Glycans mediate a plethora of transient, specific, non-enzymatic biological encounters that are sometimes termed ‘recognition events’. The rolling adhesion of leukocytes along the venule inner endothelium, for example, is orchestrated by weak glycan–protein interactions that allow local leukocyte activation and tighter interactions to form prior to extravasation (Norman *et al.*, 2000 ▸). Self/nonself distinction in innate immunity, which may trigger non-reversible immunological responses, offers other examples of transient glycan–protein complexes (Blaum *et al.*, 2015 ▸; Hansen *et al.*, 2016 ▸). Cell-bound glycans are often engaged in cell–cell communication and also in the recognition between viruses and their target cells, sometimes before other high-affinity receptors come into play. From a biophysical point of view, most glycan–protein interactions are characterized by low affinities (with *K*_d_ values of >1 µ*M*) resulting from hydrophobic interactions with aromatic amino acids, often tryptophans, and from hydrogen bonding, including structured water molecules. Such weak and therefore often transient interactions are not easy to study by means of structural biology. The saturation-transfer difference NMR (STD-NMR) experiment (Mayer & Meyer, 1999 ▸, 2001 ▸), however, works particularly well in this transient interaction range. The sole requirement is that the interaction under investigation can be approximated *in vitro* with a large protein-like entity (up to the megadalton range and higher) and a small oligosaccharide (or other small ligand) in the range of hundreds to thousands of daltons. The protein-like interaction partner could be, for example, a membrane-bound receptor (Claasen *et al.*, 2005 ▸), a virus (Benie *et al.*, 2003 ▸) or an entire cell (Claasen *et al.*, 2005 ▸). A large variety of proteins have been investigated with respect to glycan binding by STD-NMR. The list includes bacterial toxins that bind to histo-blood group antigens (Heggelund *et al.*, 2012 ▸; Vasile *et al.*, 2014 ▸), glycosyltransferases (Angulo *et al.*, 2006 ▸; Jayalakshmi *et al.*, 2004 ▸), antibodies against glycan antigens (Enríquez-Navas *et al.*, 2015 ▸; Houliston *et al.*, 2007 ▸, 2009 ▸; Tsvetkov *et al.*, 2012 ▸), galectins (Kövér *et al.*, 2010 ▸; Miller *et al.*, 2011 ▸; Yongye *et al.*, 2012 ▸), plant glycosidases (Kuntothom *et al.*, 2010 ▸), human complement factor H (Blaum *et al.*, 2015 ▸, 2016 ▸), and the innate immune receptors DC-SIGN and langerin (Mari *et al.*, 2005 ▸; Muñoz-García *et al.*, 2015 ▸; Porkolab *et al.*, 2017 ▸), among others. The STD-NMR experiment is particularly popular in the study of viruses with glycan receptors such as polyoma­viruses (see below), reovirus (Reiss *et al.*, 2012 ▸), influenza virus (Haselhorst *et al.*, 2008 ▸; Vasile *et al.*, 2014 ▸), rotavirus (Fleming *et al.*, 2014 ▸), rhinovirus (Benie *et al.*, 2003 ▸), norovirus (Rademacher & Peters, 2008 ▸) and adenovirus (Lenman *et al.*, 2018 ▸). In this article, we review mostly examples from our own work on polyomavirus capsid proteins to illustrate the synergy between crystallography and STD-NMR (Blaum *et al.*, 2015 ▸; Neu *et al.*, 2012 ▸, 2013 ▸).

## 2. Probing interactions by ‘ligand-based’ NMR   

NMR spectroscopy allows the observation of biological molecules, their interactions and dynamics in aqueous solution. Like other forms of spectroscopy (such as UV–Vis), NMR uses electromagnetic radiation to induce and probe transitions between different energy levels in molecules. In contrast to optical spectroscopy techniques, the transitions that are induced in NMR are of low overall energy, which is why radiofrequency pulses are used. As the name implies, NMR relies on the properties of atomic nuclei, namely their angular momentum (‘spin’) and coupled magnetic moment. NMR energy levels represent different nuclear spin states under the influence of a strong external field, *i.e.* the permanent NMR magnet. In the detection unit of the NMR spectrometer, tiny currents are recorded when atomic nuclear resonance frequencies (chemical shifts, in NMR terminology) are matched by the radiofrequency pulses and energy-level transitions are induced. Because resonance frequencies are highly susceptible to changes in the chemical environment, they can be used to extract structural information and characterize binding events that cause so-called chemical shift ‘perturbations’, *i.e.* changes in the chemical shifts of those amino acids whose atomic surroundings are altered by ligand binding.

A large number of studies address the interaction of protein receptors with ligands that are considerably smaller than the receptor protein (with molecular weights <1–2 kDa). In general, there are two NMR approaches that yield information on protein–ligand interactions. One approach is protein-based and monitors protein chemical shift perturbations upon ligand titration (Williamson, 2013 ▸). This approach requires stable isotope labelling of the protein receptor (usually ^15^N) and the recording of two-dimensional NMR spectra, and is limited by the size of the protein. While the ligand itself remains ‘invisible’ in chemical shift perturbation experiments, *i.e.* its chemical shifts are not being recorded, its interaction with the ^15^N-labelled protein is visualized in the form of residue-specific chemical shift changes that are subsequently used to delineate ligand-binding sites and/or allosteric binding effects. STD-NMR experiments, on the other hand, fall into the category of ‘ligand-based’ NMR experiments, in which small-molecule ligands, not proteins, are used as probes of complex formation (Meyer & Peters, 2003 ▸). In ligand-based NMR experiments the protein remains ‘invisible’ and only the ligand chemical shifts are being observed. As a consequence of the ligand detection, many shortcomings of protein-based NMR are circumvented: with STD-NMR a large molecular weight protein is not a problem, no isotope labelling is required and protein concentrations are generally in the low-micromolar range. As small ligands have a limited number of nuclei and their NMR spectra contain little spectral overlap, the resonances of the ubiquitous ^1^H nuclei (protons, in NMR terminology) can be used. Therefore, STD-NMR experiments can be conducted on any NMR spectrometer and typically require only a fraction of the measurement time compared with chemical shift perturbation experiments, as well as unlabelled protein. In favourable cases, good-quality spectra are obtained in less than 1 h. On the other hand, STD-NMR does not provide any direct structural information on the protein. Instead, a protein–ligand interaction is observed entirely from the point of view of the ligand.

## 3. STD-NMR experiments as an easy and beautiful complement to crystallography   

**Figure 1:**
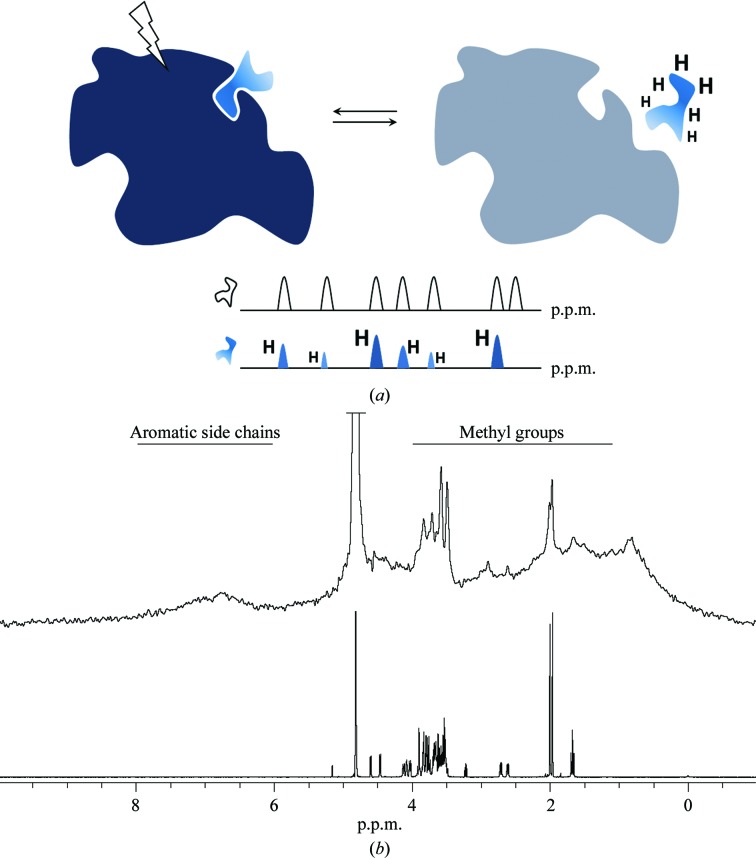
Schematic view of the STD-NMR technique. (*a*) Top: direct excitation (saturation) of the protein protons leads to rapid spin diffusion and saturation transfer to a bound small ligand (light blue). After dissociation, the large, directly excited protein returns rapidly to the ground state (grey), while the ligand maintains the proton-specific excitation pattern that was transferred during the lifetime of the complex (light blue, with differentially excited protons). Bottom: schematic representation of the free-ligand spectrum with equally intense resonance signals (top, black) and the STD-NMR difference spectrum (bottom, blue) with relative peak intensities representing the location of each ligand proton in the binding pocket of the protein. (*b*) Proton resonance spectral windows of a 150 kDa protein (top) and a 970 Da tetrasaccharide (bottom). The HDO signal at 4.8 p.p.m. is truncated in both spectra. Spectra were recorded at 288 K and re-referenced to 298 K (Clore & Potts, 2012 ▸). The relatively sharp signals in the protein spectrum at 2 p.p.m. and between 3.5 and 4 p.p.m. represent N-glycosylation chains with increased flexibility, hence their reduced linewidths.

STD-NMR relies on saturation transfer from protein proton resonances to protons of a ligand exchanging between a protein-bound and free state. In short, proton resonances of a protein are selectively saturated at radiofrequencies that are remote from any ligand resonance frequencies (Fig. 1 ▸ *a*). During the lifetime of the complex this saturation is passed on to the protons of the bound ligand. If this saturation transfer happens, the resulting proton spectrum resembles the free-ligand spectrum in terms of chemical shifts but the individual peak intensities are differentially attenuated, depending on the positioning of the ligand protons in the binding site (Fig. 1 ▸ *a*). At a very superficial level, the experiment has some similarity to the FRET experiment in that both are spectroscopic techniques in which one entity is selectively excited and energy transfer to another entity is observed only if certain requirements such as distance range or relative orientation are fulfilled. The distance cutoff for NMR saturation transfer is in the 4–5 Å range. Selective saturation of protein resonances can be achieved if the ligand chemical shift range is distinct from the protein chemical shift range, which is often the case (Fig. 1 ▸ *b*).

A qualitative match of STD-NMR data to a high-resolution structure of the corresponding complex delivers many valuable insights and strongly complements the structural data. A typical STD-NMR experiment takes just under 1 h and requires a 5–20 µ*M* aqueous solution of unlabelled protein and a 10–1000-fold excess of one or several small (potential) ligands. The typical sample volume is 200 µl (if NMR tubes with an internal diameter of 3 mm are used). Before structural information on the complex is available, the method serves as a screening tool for the identification of binding ligands from mixtures of ligand candidates. Hits are then taken to co-crystallization or crystal-soaking experiments. When structural information on protein–ligand complexes is already available, STD-NMR is a straightforward tool to test structure-based predictions, for example to test binding modes of structurally related ligands or evaluate ligand binding upon structure-based mutagenesis. Because saturation transfer between the protein and ligand is distance-dependent (roughly proportional to *r* ^−6^), a simple one-dimensional proton STD-NMR spectrum comprises information about the geometry of the complex. The relative intensities in an STD-NMR spectrum directly translate into portions of the ligand that are in close contact with the protein surface (Fig. 1 ▸ *a*). In a qualitative interpretation, relative intensities in an STD-NMR spectrum therefore deliver a binding epitope at atomic resolution (Mayer & Meyer, 2001 ▸). This is particularly useful for characterizing protein–glycan interactions because even for complex, branched oligosaccharides the method can readily elucidate those parts of the glycan that are the essential binding determinants. In glycobiology, often we are not dealing with a single ligand. Instead, a number of structurally related glycans with a common epitope can often undergo interactions with a given protein and STD-NMR is a suitable tool to analyze (or predict) the full range of glycans that contain the essential binding motif (Fiege *et al.*, 2012 ▸). As the STD-NMR spectrum contains information about ligand–protein distances in the complex, it can be used to evaluate the conclusions drawn from a crystal structure in solution, possibly aiding in resolving ambiguities arising from crystal contacts. Finally, it can provide structural restraints for hybrid refinement protocols. Thus, STD-NMR data can be directly and synergistically compared with the corresponding crystallo­graphic data, as exemplified below.

Conceptually, the physics of the STD-NMR experiment can be broken down into distinct processes that help in understanding its key features, such as the molecular-weight and *K*_d_ ranges in which the experiment performs best. Assuming that most readers prefer applicability over theory, we will now first discuss the applications of STD-NMR experiments and then return to the stepwise theoretical description of the method in the second part of this review, explaining physical concepts such as saturation and saturation-transfer mechanisms for those readers who are considering applying the method themselves.

## 4. The polyomavirus showcase   

**Figure 2:**
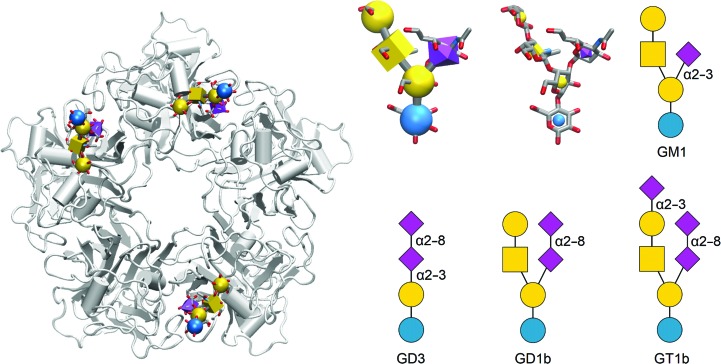
Architecture of a polyomavirus VP1 pentamer with the location of the canonical glycan-binding site and ganglioside oligosaccharides used in the text. Left: the SV40 VP1 pentamer (grey cartoon representation) with three of the five GM1 glycan-binding sites occupied (Neu *et al.*, 2008 ▸). GM1 is displayed using the 3D-SNFG (Symbol Nomenclature for Graphical Representation of Glycans) representation. Right, top row, left to right: GM1 glycan in 3D-SNFG, 3D-SNFG icon and SNFG representations. Right, bottom row, left to right: b-series ganglioside GD3, GD1b and GT1b oligosaccharides in SNFG representation. The *DrawGlycan-SNFG* server (Cheng *et al.*, 2017 ▸) and the 3*D-SNFG* script (Thieker *et al.*, 2016 ▸) for *VMD* (Humphrey *et al.*, 1996 ▸) were used to generate the SNFG-type representations.

Polyomaviruses (PyVs) are a class of small double-stranded DNA viruses that can cause mild to fatal disease (including cancer) in mammals, birds and fish. Most PyVs bind to sialyl­ated glycans that are presented on the host-cell plasma membrane, either as glycosphingolipid head groups or as glycan branches on glycoproteins. In some PyVs these glycans serve as sole receptors, while in others additional receptors have been identified. One of the best known PyVs is *Simian Virus 40* (SV40), a contaminant of early live poliovirus vaccines, which were produced in rhesus monkey kidney cells. Fortunately, SV40 is not associated with disease in humans (Sweet & Hilleman, 1960 ▸; Strickler *et al.*, 1998 ▸). The SV40 receptor is a small sialylated glycolipid, the ganglioside GM1 (Tsai *et al.*, 2003 ▸), the binding of which directly triggers membrane curvature and invagination and, eventually, cellular uptake of the virus (Ewers & Schelhaas, 2012 ▸). Each PyV capsid is constructed mainly from 360 copies of the major capsid protein VP1, which adopts the so-called jelly-roll fold (Liddington *et al.*, 1991 ▸; Stehle & Harrison, 1997 ▸; Stehle *et al.*, 1996 ▸), with varying amounts of smaller capsid proteins that coat the capsid interior. Five VP1 monomers assemble to form the pentameric VP1 capsomer, 72 of which make up the icosahedral capsid. In most PyVs of known structure, five sialic acid-binding sites are arranged symmetrically around the central fivefold axis of the VP1 pentamer (Fig. 2 ▸). Despite the overall structural conservation of the VP1 pentamer and the also often-conserved location of the sialic acid binding site between three exposed loops, the structural architecture of the binding site, the amino-acid residues present in the loops and the glycan specificity vary notably between different PyVs (Neu *et al.*, 2011 ▸, 2012 ▸; Stehle *et al.*, 1994 ▸; Ströh *et al.*, 2015 ▸). While the nonreducing-end sialic acid (Neu5Ac) ‘cap’ forms the majority of contacts in most PyVs characterized to date, a few additional interactions outside the Neu5Ac coordination confer varying glycan specificities that are reflected in differing glycan microarray profiles, haemagglutination patterns and receptor usage in transduction experiments (Neu *et al.*, 2008 ▸, 2012 ▸, 2013 ▸). While SV40 binds the branched, monosialylated GM1 pentasaccharide (Fig. 2 ▸), the human PyVs BKPyV (which can cause nephropathy in kidney-transplant patients) and Merkel cell PyV (MCPyV, an aetiological agent of Merkel cell carcinoma) do not bind to GM1 (Erickson *et al.*, 2009 ▸; Low *et al.*, 2006 ▸). Instead, BKPyV specifically engages α2–8 disialylated gangliosides such as GD3 and more complex gangliosides that contain the linear GD3 tetrasaccharide as part of a branched glycan structure (for example the GD1b and GT1b glycans; see Fig. 2 ▸; Low *et al.*, 2006 ▸; Neu *et al.*, 2013 ▸). The functional receptor for MCPyV has remained unknown to date, but it has been shown that MCPyV VP1 pentamers bind the GT1b ganglioside and shorter, linear sialylated glycans containing α2–3-linked Neu5Ac (Erickson *et al.*, 2009 ▸; Neu *et al.*, 2012 ▸).

Crystallographic analysis of the capsid–glycan interaction in the PyV family is relatively straightforward owing to an N- and C-terminally truncated VP1 construct that can be produced in *Escherichia coli* and forms VP1 pentamers that are unable to assemble into full capsids, with an approximate weight of 150 kDa (Stehle & Harrison, 1997 ▸; Fig. 2 ▸). While some of the exposed glycan-binding sites in the VP1 pentamer might be inaccessible owing to crystal contacts, the presence of five identical sites in a VP1 pentamer nevertheless allowed the determination of VP1–glycan complexes by crystallography. In order to define differences in glycan specificity between different polyomaviruses, we performed X-ray crystallography and STD-NMR spectroscopy on a number of PyV VP1–glyan complexes, elucidating the structural diversity of glycan binding within this virus class (Neu *et al.*, 2011 ▸, 2012 ▸, 2013 ▸; Ströh & Stehle, 2014 ▸; Ströh *et al.*, 2015 ▸).

### 4.1. BKPyV: finding the correct binding conformation despite crystal contacts   

**Figure 3:**
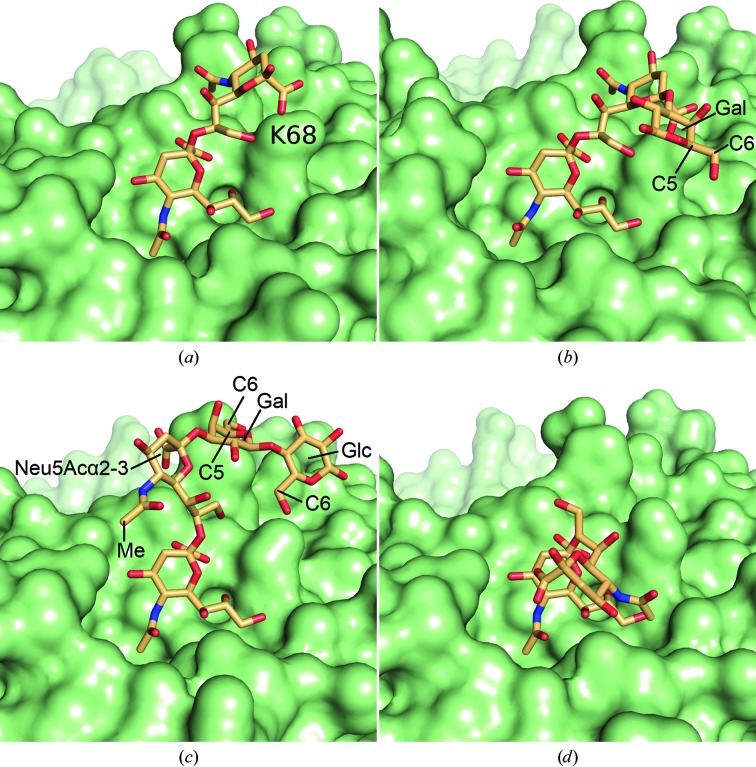
The four GD3 glycan-binding sites as seen in the BKPyV VP1–GD3 crystal (Neu *et al.*, 2013 ▸). Only the GD3 orientation seen in (*a*) and (*b*) is compatible with the STD-NMR difference spectrum shown in Fig. 4 ▸. Residue Lys68 is highlighted in (*a*). C atoms for which the attached protons are explicitly discussed in §[Sec sec4.1]4.1 are labelled where modelled.

**Figure 4:**
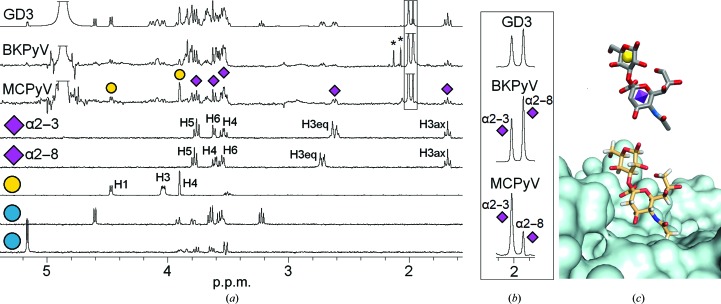
Distinct binding epitopes of MCPyV and BKPyV VP1 in complex with the GD3 glycan. (*a*) GD3 reference spectrum (top), BKPyV and MCPyV VP1 STD-NMR difference spectra (second and third from top) and TOCSY spectra of individual pyranoses in the GD3 tetrasaccharide, labelled with SNFG representations as in Fig. 2 ▸. TOCSY spectra for both anomeric forms of the reducing-end Glc ring are shown (bottom and second from bottom). Impurities are represented by asterisks. Selected resonances in the TOCSY spectra are labelled. The H3–H6 proton resonances of the Neu5Acα2–3 ring and the H1 and H4 proton resonances of the Gal ring are clearly recognizable in the MCPyV VP1 STD difference spectrum (at 4.48 and 3.90 p.p.m., respectively). The epitope seen in the BKPyV VP1 difference spectrum, in contrast, mostly includes resonances of both Neu5Ac rings. The truncated peaks at 2 p.p.m. (framed) belong to the Neu5Ac methyl groups, which are also shown in full in (*b*). HDO and methyl-group signals were truncated. More detailed assignments of both STD difference spectra can be found in Neu *et al.* (2012 ▸, 2013 ▸). NMR spectra were recorded at 283 K and re-referenced to 298 K; the STD saturation time was 2 s. (*c*) 3D-SNFG (top, in grey) representation of the GD3 epitope as bound in the MCPyV VP1–GD3 crystal structure (bottom, with the GD3 middle disaccharide in light orange; Neu *et al.*, 2012 ▸). Aliphatic H atoms were added to the crystal structure in *PyMOL*.

In the crystal structure of the complex between BKPyV VP1 and the GD3 tetrasaccharide, considerable differences exist between the four ligand molecules within the crystallo­graphic asymmetric unit (Fig. 3 ▸). The GD3 glycan is disialylated with a Neu5Acα2–8Neu5Acα2–3 ‘cap’ at the nonreducing end (Fig. 2 ▸). While the terminal (nonreducing end) Neu5Acα2–8 ring of GD3 adopts essentially the same orientation within each of the four occupied binding sites, three different orientations of its glycosidic linkage are observed, leading to remarkably different orientations of the second Neu5Acα ring from the reducing end (the Neu5Acα2–3 ring). In addition, electron density is observed for only two pyranoses in two binding sites, for three rings in another site and for the full GD3 tetrasaccharide in the fourth occupied binding site. The many and conserved interactions between BKPyV VP1 and the terminal Neu5Acα2–8 are reflected in the prominent role of this monosaccharide in the STD-NMR difference spectrum (Fig. 4 ▸). Interestingly, some ligand orientations observed in the crystal structure are not compatible with the STD difference spectrum of this complex. For those two binding sites in which sufficient electron density allowed model building of the Gal ring (Figs. 3 ▸ *b* and 3 ▸ *c*), its H5 and H6 protons fall within ≤4 Å of the nearest aliphatic C atom with the protein, meaning that good saturation transfer to these protons would be expected. The same holds true for the H6 protons of the Glc ring within the binding site that supported modelling of the full GD3 glycan (Fig. 3 ▸ *c*). However, none of these protons produced strong peaks in the BKPyV VP1–GD3 STD difference spectrum (Fig. 4 ▸), rendering the conformations less likely to be the true binding conformations. Additionally, only one of the three Neu5Acα2–8Neu5Acα glycosidic linkage orientations (Figs. 3 ▸ *a* and 3 ▸ *b*) is fully compatible with the NMR spectrum, while the other two orientations (Figs. 3 ▸ *c* and 3 ▸ *d*) are ruled out. In one of the latter orientations (Fig. 3 ▸ *c*) the strongly observable Neu5Acα2–3 ring methyl group would not be expected in the STD spectrum as its protons would be further than 5 Å from the nearest aliphatic protein C atom. In the other orientation (Fig. 3 ▸ *d*) the H5 and H6 protons would not be expected in the STD spectrum for the same reason, but the spectrum contains contributions from both protons (Fig. 4 ▸). Careful evaluation of the crystal lattice (not shown) also supports the notion that only one of the four ligand-binding sites in the crystal truly reflects GD3 glycan recognition by BKPyV VP1 (that shown in Fig. 3 ▸ *a*), while the other three sites reflect crystal-packing bias [in the binding site shown in Fig. 3 ▸(*b*) only insofar as the Neu5Acα2–3Galβ linkage suffers reduced flexibility but no distortion of the Neu5Acα2–8Neu5Acα linkage occurs].

### 4.2. BKPyV and MCPyV: one ligand, two epitopes   

A comparison of our STD-NMR spectra highlights the existence of individual epitopes with which each virus recognizes its respective glycan ligand. For instance, a comparison of the MCPyV and BKPyV VP1 STD-NMR difference spectra with the GD3 tetrasaccharide revealed that both proteins bind to a different subset of pyranoses within this glycan (Fig. 4 ▸). While in the BKPyV VP1–GD3 STD difference spectrum resonances of the nonreducing-end disaccharide moiety of GD3, Neu5Acα2–8Neu5Acα, are most prominent (Fig. 4 ▸), the MCPyV VP1–GD3 spectrum shows another epitope comprising instead the GD3 ‘middle’ disaccharide Neu5Acα2–3Galβ (Fig. 4 ▸). In particular, the Gal H4 and the equatorial Neu5Acα2–3 H3 resonances are prominent in the MCPyV STD difference spectrum but are absent in the BKPyV difference spectrum (Fig. 4 ▸). In addition, the Neu5Acα2–3 methyl-group peak is strong in the MCPyV difference spectrum (Fig. 4 ▸). In the respective BKPyV spectrum both the Neu5Acα2–3 and Neu5Acα2–8 methyl-group peaks are prominent and the equatorial Neu5Acα2–8 H3 resonance is more intense than the Neu5Acα2–3 H3 resonance, suggesting a more important role for the terminal Neu5Acα2–8 ring and a minor role for the Gal ring in the BKPyV-bound GD3 epitope. No strong contributions from the reducing-end Glc ring are observed in either spectrum. These observations are in perfect agreement with the respective complex crystal structures. In the MCPyV VP1–GD3 crystallographic model electron density was only observed for the Neu5Acα2–3Galβ di­saccharide, and the orientation of this disaccharide within the binding pocket fits well with the NMR data. In particular, the bound GD3 conformation brings the Neu5Acα2–3 methyl group, H4 and equatorial Neu5Acα2–3 H3, as well as the Gal H4, which have prominent resonances in the STD difference spectrum, into close proximity to the protein (Fig. 4 ▸).

### 4.3. SV40 and BKPyV: a point mutation causes a receptor switch   

**Figure 5:**
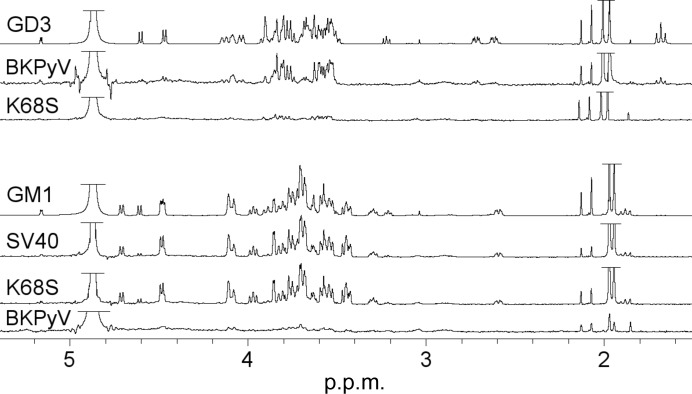
The K68S mutation in the BKPyV VP1 changes the glycan specificity from the GD3 to the GM1 glycan. Top three spectra: GD3 ^1^H reference spectrum, wild-type BKPyV VP1–GD3 glycan STD-NMR difference spectrum and K68S mutant BKPyV VP1–GD3 STD-NMR difference spectrum. Bottom four spectra: GM1 ^1^H reference spectrum, SV40 VP1–GM1 glycan STD-NMR difference spectrum, K68S mutant BKPyV VP1–GM1 STD-NMR difference spectrum and wild-type BKPyV VP1–GM1 glycan STD-NMR difference spectrum. HDO and methyl-group signals were truncated. NMR spectra were recorded at 283 K and re-referenced to 298 K; the STD saturation time was 2 s.

The sequence identity between the human BKPyV and monkey SV40 VP1 proteins is 74%. Despite their different glycan specificities (disialylated gangliosides in BKPyV *versus* GM1 in SV40), the architecture of the respective glycan-binding sites of the two viruses is similar. In particular, the orientation of the nonreducing-end Neu5Ac ring is identical in both binding sites and is determined by a similar set of hydrophobic and polar interactions (Neu *et al.*, 2013 ▸). In the SV40–GM1 glycan complex, the only additional directed interaction with any of the other four pyranoses in GM1 is a hydrogen bond between the Ser68 side chain and a Gal hydroxyl group at position C6 of the nonreducing-end Gal that caps the second arm of the branched pentasaccharide (Neu *et al.*, 2008 ▸). In BKPyV VP1, residue 68 is a lysine whose side chain coordinates the second Neu5Ac ring carboxy group in the GD3 (GD1b…) glycan (Fig. 3 ▸ *a*). Here, this is again the only directed contact observed for this ring. This observation led us to attribute the different glycan specificities of SV40 and BKPyV to VP1 amino acid 68 alone and to hypothesize that replacement of the BKPyV VP1 Lys68 with serine (BKPyV K68S) would enable this mutant virus to use GM1, not GD3 and other gangliosides with disialylated ‘caps’, as a receptor. Indeed, the designed mutant virus was found to bind GM1 in cell culture, and the respective VP1 pentamer yielded a glycan microarray binding profile that was significantly altered by the mutation (Neu *et al.*, 2013 ▸). Crystal structure determination was not attempted for the BKPyV K68S VP1–GM1 glycan complex, but an STD-NMR spectrum was obtained and compared with the wild-type BKPyV and SV40 VP1 spectra. To our surprise, the STD-NMR difference spectrum of the K68S mutant not only showed clear binding to the GM1 glycan but was indistinguishable from the reference SV40 VP1–GM1 glycan spectrum, while GD3 binding was no longer observed (Fig. 5 ▸). Generally, the absence of visible saturation transfer can be caused by nonbinding as well as by slow off-rates (see §[Sec sec9]9), and hence it is difficult to draw a definite conclusion as to whether an interaction takes place from a ‘negative’ STD-NMR difference spectrum alone, which is a clear limitation of the method. However, it appears reasonable to assume that the loss of previously observable saturation transfer upon structure-based mutagenesis of an essential amino acid is more likely to be representative of impaired binding than of a reduced off-rate.

## 5. A nonpolar interactions filter   

The protein ‘fingerprints’ shown in Figs. 4 ▸ and 5 ▸, and especially the striking similarity of the SV40–GM1 and BKPyV K68S–GM1 spectra, highlight an aspect of ligand–protein complexes that is often underestimated from the analysis of crystal structures alone: hydrophobic interactions. While high-resolution (<2.0 Å) crystal structures of protein–glycan complexes permit straightforward analysis of free-energy contributions from hydrogen bonds and salt bridges, contributions from nonpolar interactions are more difficult to extract (let alone quantify) from purely visual analysis. Some of the polar interactions observed in complex crystal structures replace interactions similar to those provided by ordered water molecules in the solvated unliganded protein under investigation, a factor with both enthalpic and entropic parts, whose net contributions to the free energy of complex formation are difficult to estimate. Despite the many hydroxyl and other polar groups in pyranoses, most carbohydrates have a non­polar face and, consequently, protein–glycan complex formation can be rationalized with the hydrophobic effect. Recently, nonconventional CH⋯O hydrogen bonds in carbohydrate–carbohydrate and carbohydrate–protein complexes have received increased attention (Aeschbacher *et al.*, 2017 ▸; Zierke *et al.*, 2013 ▸), and the over-representation of aromatic side chains in glycan-binding pockets and associated CH–π interactions has been well documented (Hudson *et al.*, 2015 ▸; Ramírez-Gualito *et al.*, 2009 ▸). Here, STD-NMR spectra complement a natural shortcoming of crystallography because most STD-NMR analyses are limited to atoms that are invisible in the large majority of X-ray data sets: protons. More precisely, because the spectra are collected in aqueous, *i.e.* polar, buffers, only aliphatic ligand proton resonances are visible, while the polar/acidic set of H atoms in glycans (those that are part of hydroxyl, amine and carboxy groups) rapidly exchange with the surrounding solvent molecules. As a consequence, exactly those polar interactions that are readily picked up from visual inspection of crystal structures are missing from STD-NMR spectra. Instead, the spectra provide information exclusively gained from the aliphatic protons, which do not exchange with water molecules at significant rates and which are not visualized by X-ray crystallography.

## 6. Structure refinement based on STD-NMR   

STD-NMR spectra deliver individual ‘fingerprints’ of transient protein–ligand encounters, and the STD intensities of individual proton resonances may serve as restraints in structure-refinement protocols. In fact, the physical principles that govern spin diffusion (see below) and other forms of NMR-typical energy exchange in proteins and ligand–protein complexes are well understood and can be described analytically by the complete relaxation and conformational exchange matrix (CORCEMA) theory (Rama Krishna & Jayalakshmi, 2008 ▸; Moseley *et al.*, 1995 ▸). In NMR terminology, ‘conformational exchange’ includes complex formation and dissociation. The CORCEMA theory was originally developed for the analysis of two-dimensional NOESY spectra of interacting molecules and was later extended to saturation-transfer (ST) experiments (Jayalakshmi & Rama Krishna, 2002 ▸). With the *CORCEMA-ST* software, which runs on *MATLAB*, STD peak intensities can be used not just to semi-quantitatively classify results from crystallography and NMR as roughly agreeing on the same ligand-binding epitope, as in our PyV examples, but to quantitatively scrutinize bound ligand conformations in crystallographic models. This can be performed *via* the prediction of STD intensities and buildup curves from crystallographic complex models and quantitative comparison with experimental STD-NMR spectra (for a user-friendly *CORCEMA-ST* protocol, see Enríquez-Navas *et al.*, 2015 ▸). STD intensities can also be fed into hybrid refinement protocols that aim to improve docking results or other means of initial ligand–protein structure generation based on apo protein structures and some sort of conformational knowledge of the ligand *via* experimentally restrained simulated-annealing torsion-angle optimization (Jayalakshmi & Rama Krishna, 2004 ▸).

## 7. Extending structural biology to large and complex systems   

To date, a major drawback of high-resolution techniques in structural biology is the requirement for highly purified and homogeneous samples. For multi-domain proteins, truncated constructs are often easier to crystallize, but the findings from such constructs are best scrutinized in the context of the full protein. The same holds true for multi-component systems, which are often dissected into smaller parts for crystallo­graphic studies following a ‘divide-and-conquer’ strategy. Taking a protein out of its biological context can, however, lead to its functional inactivation. This is especially problem­atic for membrane proteins, which are often solubilized in detergents prior to structural studies or re-integration into liposomes. In both cases, STD-NMR can fill experimental gaps because the ‘protein’ part of the NMR sample can in fact be a complex, inhomogeneous, protein-containing entity that would itself resist high-resolution techniques. STD-NMR spectra have been recorded using intact viruses or virus-like particles with glycan receptors (Benie *et al.*, 2003 ▸; Haselhorst *et al.*, 2008 ▸; Rademacher & Peters, 2008 ▸). We made use of the technique to screen for potential oligosaccharide ligands in a multi-glycosylated 20-domain protein with 19 linkers of varying flexibility and took the results to two-domain constructs for crystallization (Blaum *et al.*, 2015 ▸). In fact, STD-NMR spectra can be recorded using live mammalian cells as the selectively excited component (Claasen *et al.*, 2005 ▸; Mari *et al.*, 2005 ▸; Vasile *et al.*, 2018 ▸). A combination of unmodified cells and cells that overexpress a specific membrane protein (or, conversely, harbour a knockdown of one) can yield conclusive results for small-molecule binding to a membrane-embedded protein (Vasile *et al.*, 2018 ▸). A similar strategy was used, for example, to assess the functional integrity of liposome-integrated integrins: STD-NMR spectra on such liposomes and an integrin-binding peptide were recorded and compared with spectra obtained with native platelets (Claasen *et al.*, 2005 ▸). Alternatively, the complexity of the system under investigation can also stem from the ‘buffer’ rather than the selectively excited entity. For example, the detection and epitope characterization of anti-GM1 antibodies *via* STD-NMR directly in serum obtained from patients with Guillain–Barré and Fisher syndromes after supplementation with the GM1 glycan has been demonstrated (Houliston *et al.*, 2007 ▸). At such a level of sample complexity, an additional but simple ‘filter’ may be employed: while the excitation may still be selective insofar as no ligand resonances are excited (which can be checked with a separate sample that contains only the ligand), the excitation may not be selective with respect to a single protein and the resulting STD-NMR difference spectrum may contain signals that derive from additional interactions with the ligand under investigation or with other small molecules in solution. This problem can, however, be overcome simply by the subtraction of STD-NMR difference spectra obtained using controls, for example a cell suspension without the overexpressed membrane-bound protein of interest or serum spectra without the ligand in question added, yielding so-called saturation-transfer double difference (STDD; Blaum *et al.*, 2015 ▸; Claasen *et al.*, 2005 ▸). Alternatively, a cell type that does not, in its native form, express the membrane protein under investigation at all can be used. Human and avian influenza virus strain haemagglutinin expressed in 293T cells, for example, could be studied with respect to an influenza virus receptor part, Neu5Acα2–6Galβ1–4GlcNAc, without any cellular background (Vasile *et al.*, 2018 ▸). Needless to say, STD-NMR experiments can also serve to assess the quality of a given protein batch after liposome reconstitution or refolding when spectra are recorded under the same conditions as a well characterized reference batch and STD intensities are compared. Similar to quality control by any ligand-binding assay, the percentage of folded/active protein in a given preparation can thus be a assessed by STD-NMR (Da Veiga *et al.*, 2016 ▸).

## 8. More complex variants of STD-NMR   

STD-NMR is, of course, amenable to more complex setups: selective excitation is, for example, also achievable upon isotope labelling of one partner in mixtures in which both interaction partners are more alike in terms of their proton resonance frequency range than in the protein–glycan scenario, or to achieve differential pre-saturation (Kövér *et al.*, 2007 ▸, 2010 ▸; Wagstaff *et al.*, 2010 ▸). The method can also be extended to multi-dimensional spectra (Wagstaff *et al.*, 2010 ▸; Mayer & Meyer, 2001 ▸). Because the interaction with a large protein serves as a sort of spectral filter, STD-NMR can be used to screen for binding with mixtures of potential ligands, to determine dissociation constants (Angulo *et al.*, 2010 ▸) and complex binding isotherms (Mallagaray *et al.*, 2017 ▸), or to evaluate the competition of two ligands for a common binding site and determination of IC_50_ values (Mayer & Meyer, 2001 ▸). There is even an extended version of the experiment in which the relative binding poses of two independent ligand types to a given protein are correlated *via* magnetization transfer first from one ligand to a protein and, subsequently, to another ligand type of the same protein (Orts *et al.*, 2008 ▸).

## 9. The STD-NMR experiment explained step by step   

Conceptually, what happens during the STD-NMR experiment from a more physical point of view can be divided into different steps, which are outlined in the following.

### 9.1. Selective excitation of the protein and saturation   

At the beginning of the experiment, selective excitation utilizing a cascade of Gaussian-shaped radiofrequency pulses is employed for a period of up to several seconds (saturation time). Application of such a pulse cascade leads to saturation (also called pre-saturation), reflecting a state of the spin system where no more energy is absorbed since relaxation processes can no longer outweigh the influx of energy. Essentially, in this state spin populations are equalized and respective transitions are no longer observable. Selective saturation in STD-NMR is achieved by tuning the radiofrequency irradiation to a spectral window that is populated only by resonances of the receptor protein. Typical spectral windows for selective saturation of the protein are found in the range 1–4 p.p.m. (protein methyl groups in alanine, valine, leucine, isoleucine, methionine and threonine) or in the aromatic and NH region of 6–8 p.p.m. (phenylalanine, tyrosine and tryptophan side chains), assuming that no ligand resonances are present in at least one of these spectral regions (Fig. 1 ▸ *b*).

### 9.2. Spin diffusion and saturation transfer   

During the saturation time, a non-equilibrium situation is created for the selectively excited spins (because equal energy-level populations are not in agreement with the Boltzmann distribution). As in other forms of spectroscopy, the system attempts to relax back to the ground state and therefore energy needs to dissipate from the excited state. For nuclear spin systems this excess energy is passed between spins that are close in space, irrespective of chemical bonds. In large proteins slow molecular tumbling renders these through-space effects (cross-relaxation) very efficient, and non-equilibrium magnetization acquired through the saturating pulse train quickly spreads out from the selectively excited side-chain protons across the entire molecule in a process called spin diffusion (dark blue colour in Fig. 1 ▸ *a*, left). If the protein forms a complex with a ligand whose proton resonances were not initially directly excited (for example a small oligosaccharide), cross-relaxation takes place between protons in the ligand-binding site and the protons of the ligand. Thus, the non-equilibrium magnetization is transferred from the protein to the ligand whilst residing in the binding pocket (blue ligand in Fig. 1 ▸ *a*, left). Spin diffusion strongly depends on the overall size (tumbling rate) of the molecule. For larger proteins non-equilibrium magnetization is rapidly lost (grey colour in Fig. 1 ▸ *a*, right) whereas for a small ligand, after dissociation from the complex, the non-equilibrium state is conserved for a much longer period of time. Assuming fast dissociation of the protein–ligand complex, this phenomenon allows observation of the non-equilibrium state using the resonance lines of the free ligand (blue ligand in Fig. 1 ▸ *a*, right). In other words, the STD-NMR experiment first takes advantage of the large size of a receptor protein, allowing efficient saturation transfer to a small ligand, and then engages the small size of the free ligand to benefit from the long lifetime of the non-equilibrium magnetization (saturation) in the unbound state of the ligand. Large ligand:protein ratios are employed in order to ensure that many ligands sample the binding site in a given amount of time, allowing the accumulation of a maximum amount of saturated ligands and improved signal to noise. Also, from this description it is clear that a large-sized protein is beneficial in STD-NMR experiments, much in contrast to protein-based NMR experiments. In fact, the experiment actually performs better with a protein of 200 kDa or larger than with a 20 kDa protein.

If a protein–ligand complex is tight, *i.e.* the off-rate is so slow that the dissociation rate is smaller than the chemical shift differences between bound and free ligand, separate resonance lines are observed in, for example, chemical shift perturbation experiments and it is said that exchange is slow on the chemical shift timescale. For the vast majority of cases this implies that saturation received in the bound state is not observable using free-ligand ^1^H resonances. In this case, a bound ligand is essentially part of a large entity (the complex), tumbling at a slow rate and thus adopting the characteristics of a large molecule: fast decay of magnetization and line broadening to the degree that individual resonances may become invisible. Therefore, it is sometimes impossible to say whether a negative STD-NMR experiment signifies no binding or tight binding. If a weakly binding ligand is available, loss of the STD signal of the weak ligand upon addition of the tight binder can be conclusive (Blaum *et al.*, 2014 ▸). Direct STD effects are observed in the 10^−3^ and 10^−7^ *M* *K*_d_ range (Krishna & Jayalakshmi, 2008 ▸), assuming a diffusion-limited on-rate.

### 9.3. The difference spectrum   

In practice, an STD-NMR experiment comprises two slightly different NMR spectra that are recorded in a pairwise fashion: a so-called on-resonance spectrum, in which the selective excitation frequency is set on the methyl or aromatic resonances of the protein as described above, and a so-called off-resonance spectrum, with a selective frequency far outside the spectral window of any of the two components. Thus, in the off-resonance spectrum effectively no pre-saturation is achieved (but the small sample-heating effects from the radiofrequency pulses are identical to those from the off-resonance spectrum), yielding an ordinary, unbiased NMR spectrum of the mixture. If the protein is very large (for instance a whole virus) no protein resonances are observed in this spectrum simply because of the excessive line broadening; thus, the off-resonance spectrum may look mostly like an unbiased free-ligand spectrum. For the analysis, the on-resonance spectrum is subtracted from the off-resonance spectrum. The reason for this procedure is that the saturation-transfer effect is generally small and therefore more readily visualized as a difference spectrum. The off-resonance spectrum serves as a reference in the quantification of the STD effect *via* the so-called amplitude factor (Mayer & Meyer, 2001 ▸).<!?tpb=-12pt>

## 10. How to perform STD-NMR spectroscopy: practical considerations, advice and conclusions   

There are a few challenges and considerations for a structural biologist starting to use STD-NMR spectroscopy to look at protein–carbohydrate complexes. Firstly, of course, structural biologists do not usually have an NMR instrument at hand or do not know how to operate it. This is actually a rather small hurdle because the instrumentation present in the analytical section of any (synthetic) chemistry department can be used for the experiments, and usually has a service associated with its operation. Signal to noise and spectral resolution increase with field strength, but 400–500 MHz instruments will do if higher fields are not available. The software used to operate NMR spectrometers is delivered with a set of standard experiments (radiofrequency pulse sequences) and, at least for Bruker instruments, this set contains STD experiments.

One important aspect to consider before using the standard pulse sequences for protein–carbohydrate interactions is water suppression. Being by far the most concentrated component in any liquid biological NMR sample, water resonances are routinely suppressed in biological NMR applications and the respective pulse sequences. When working with carbohydrates, this fact needs to be considered carefully: water-suppression techniques are not selective for the substance; rather, they suppress the water proton resonance frequency range. Carbohydrates, unfortunately, tend to contain protons that resonate in proximity to the water proton frequencies, notably anomeric protons (Figs. 4 ▸ and 5 ▸). When water suppression is used, these signals are also suppressed, at least partially, along with the water resonances, and disappear from the spectrum or are artificially reduced. This problem can be overcome by the use of highly pure deuterated water instead of H_2_O (5–10% D_2_O is also used in water-based NMR samples for technical reasons) and removal of the water-suppression scheme from the pulse sequence. Therefore, the carbohydrate and protein need to be dissolved or buffer-exchanged into a buffer solution based on highly pure D_2_O. Buffer exchange is most feasible in spin concentrators or spin columns, as dialysis might use too much expensive D_2_O.

Another important issue is the composition of the buffer: most biological buffer substances contain proton resonances that overlap with oligosaccharide (and other small-molecule ligand) resonances and are present in a large excess over the ligand. This can lead to ugly subtraction artefacts in the STD-NMR difference spectrum right where the important ligand resonances are. Again, this hurdle is best overcome by choosing other chemicals as buffers, namely those that do not contain aliphatic protons (the favourite buffer of the NMR spectroscopist is phosphate) or are quantitatively deuterated, such as HEPES (d_18_) or Tris (d_11_), which are more expensive than phosphate. Taken together, the initial expense for a structural glycobiology laboratory moving into STD-NMR is very manageable. While highly pure D_2_O and deuterated buffers are expensive, NMR tubes (3 mm internal diameter MATCH tubes) and phosphate are cheap.

Last, but not least, it is important to know what the resonances in your STD-NMR spectrum are. Assignments for a variety of oligosaccharides and other small molecules can be found in databases and in the literature, but care should be taken when buffer conditions and temperature are varied because proton resonances are very sensitive to such changes. If you cannot find a resonance assignment for your favourite ligand, you may need to ask your favourite NMR spectroscopist for help. Generally, for small ligands a series of one-dimensional spectra such as ^1^H TOCSY and ^1^H COSY spectra as shown in Fig. 4 ▸ are sufficient, each of which takes less than a minute to record. If ^13^C resonances are required to resolve assignment ambiguities owing to signal overlap in the ^1^H dimension and ^13^C labelling is not possible, a 1 m*M* ligand sample and a few hours of measurement time are required to record ^1^H, ^13^C HSQC or HMBC spectra on the naturally occurring percentage of ^13^C nuclei (1.1%).

Irrespective of the system that you are working on, and irrespective of whether the STD experiment will work for that particular system, there is another useful aspect associated with trying the experiment: have you ever wondered whether your precious glycan or other, maybe terribly expensive, ligand is what its label says? Or how pure it might be? NMR will tell you.
